# Patient-Reported Outcome Measures for Patients with Diabetes Mellitus Associated with Foot and Ankle Pathologies: A Systematic Review

**DOI:** 10.3390/jcm8020146

**Published:** 2019-01-27

**Authors:** Ana Belen Ortega-Avila, Pablo Cervera-Garvi, Laura Ramos-Petersen, Esther Chicharro-Luna, Gabriel Gijon-Nogueron

**Affiliations:** 1 Department of Nursing and Podiatry, Faculty of Health Sciences, University of Malaga, Arquitecto Francisco Penalosa 3, Ampliación de Campus de Teatinos, 29071 Malaga, Spain; pcervera@uma.es (P.C.-G.); lauraramos.94@hotmail.com (L.R.-P.); gagijon@uma.es (G.G.-N.); 2 Department of Behavioral Sciences and Health, Miguel Hernández University, San Juan de Alicante, 03550 Alicante, Spain; ec.luna@goumh.umh.es; 3 Instituto de Investigación Biomédica de Málaga (IBIMA), 29010 Málaga, Spain

**Keywords:** diabetes mellitus, foot, ankle, psychometrics, patient-reported outcome measures, systematic review

## Abstract

Background: Diabetes mellitus (DM) is a chronic and complex disease, which is a major cause of morbidity and mortality and affects all age groups. It commonly produces secondary effects on the foot, often making daily activities impossible. Patient-reported outcome measures (PROMs) provide a standardised method of obtaining patients’ outlooks on their functional status and wellbeing. Although many instruments have been proposed for obtaining data on persons with DM whose feet are affected by the disease, in many cases the psychometric properties of the instrument have yet to be established. The principal objective of our review was to identify PROMs specific for patients with DM affecting the foot and ankle and to evaluate the psychometric properties and methodological quality of these instruments. Methods: In this systematic review, we investigate studies (published in English or Spanish) based on the use of one or more PROMs specific to foot and ankle pathologies for patients with DM (type I or II). To do so, the databases PubMed, Scopus, CINAHL, PEDro and Google Scholar were searched for studies that analysed psychometric or clinimetric properties in this respect. These were assessed according to Terwee or COSMIN criteria. Results: Of the 1016 studies identified in the initial search, only 11 were finally included in the qualitative review. Analysis according to Terwee and COSMIN criteria showed that the Foot Health Status Questionnaire (FHSQ) presented the greatest number of positive values. Conclusions: The FHSQ is the highest-quality PROM currently available for the foot and ankle, for patients with DM.

## 1. Introduction

In 2014, according to the World Health Organization (2018), 422 million adults suffered from DM worldwide, and its prevalence had almost doubled since 1980, rising from 4.7% to 8.5%. The disease, although non-communicable, is taking the form of a global epidemic and poses a growing threat to both affluent and non-affluent societies [1]. It is both chronic and complex [2], and is currently the world’s leading cause of morbidity and mortality, affecting all age groups [3].

DM is subdivided into several types, but Types I and II (TIDM and TIIDM) are the most prevalent [4]. During the course of the disease, patients may be significantly affected in terms of reduced physical activity, psychological consequences and chronic clinical complications, requiring significant medical care [5]. In the majority of cases, its symptoms are associated with localised complications, especially in the foot, and approximately 15% of patients eventually develop a diabetic foot ulcer [6].

The diabetic foot syndrome (DFS) is characterized by the presence of infection and/or ulcer, and/or deep tissue destruction as a result of underlying neuropathy and different severity ischemia from peripheral vascular disease. Diabetic patients have up to a 25% lifetime risk of developing diabetic foot syndrome [7].

Symptomatic Peripheral Artery Disease (PAD) is about twice as common among patients with diabetes mellitus than in those without. In patients with diabetes, for every 1% increase in haemoglobin A1c level, there is a corresponding 26% risk increase for PAD. PAD is more aggressive in patients with diabetes, as compared with non-diabetics. Earlier large vessel involvement and more distal symmetrical neuropathy occur in PAD. The need for major amputation is five to ten times higher in patients with diabetes than in those without diabetes [8].

The International Diabetes Foundation has termed the disease a substantial threat to public health, affecting not only patients but also their families. Patients often lose sensitivity in the extremities, especially the feet, which may result in ulceration, difficulty in walking, amputation (the most devastating complication of DFS). It is estimated that 5% to 15% of patients with foot ulcers will require amputation, with major lower limb amputations accounting for 50% of these amputations. Over 50% of the amputees will undergo another amputation within five years, (of whom 50% will not survive the next five years) [7], prolonged hospital stays and recurrent ulcers, producing high treatment costs and causing major difficulties for public health systems. These factors may also make patients less able to work, provoke early retirement and lead to difficulty in performing the activities of daily life, generating feelings of helplessness, frustration, vulnerability and a poor body image [9].

Many outcome measures have been developed to assess the status of patients with DM, including the Diabetes Management Self-Efficacy Scale (DMSES) [10], the Patient-Perceived Difficulty in Diabetes Treatment Scale (PDDT) [11], Environmental Barriers to Diabetes-Regimen Adherence (EBAS) [12], the Diabetes Health Profile for DM Type II (DHP-18) [13], the Diabetes Distress Scale (DDS) [14], the Summary of Diabetes Self-Care Activities Measure (SDSCA) [15] and the Personal Diabetes Questionnaire (PDQ) [16].

In addition, various systematic reviews have been conducted on patient-reported outcome measures (PROMs) for DM, such as Corathers et al. (2017) for pyschosocial PROMs in children and adolescents with DM [9], and Breslow et al. (1987) on the psychometric properties and theoretical grounding of instruments for evaluating self-care in persons with TIIDM [17]. Other studies have addressed the question of PROMs for patients with ankle/foot pathologies, but not specifically related to DM [18]. However, to our knowledge no previous review has been made of PROMs for patients with DM with specific reference to its impact on the foot and ankle, despite this extremity being the most commonly affected by DM and subject to major complications.

In view of these considerations, the main aim of the present review is to identify PROMs that are specific for DM affecting the foot and ankle and to evaluate their psychometric properties and methodological quality.

## 2. Material and Methods

### 2.1. Design

A systematic review was carried out to evaluate PROMs specific to the foot and ankle for patients with DM. Review registration number: CDR42019115078.

### 2.2. Search Strategy

Studies were selected for analysis, in accordance with the PRISMA guidelines [19], from a search carried out on the following databases: PubMed, Scopus, CINAHL, PEDro and Google Scholar. No time limits were imposed on the search. The search was concluded in September 2018. The search strategy obtained all the psychometric properties described by Terwee et al. [20], including construct search (patient-reported outcomes specific to the foot and ankle), population search (diabetes mellitus), instrument search (questionnaires, scales, test), measurement properties and exclusion filters.

The following search terms were used, together with the operators “OR” and “AND”: diabetes mellitus, patient-reported outcomes, foot, feet, ankle, pain, disability, funct*. (Supplementary Materials).

### 2.3. Inclusion Criteria

- Types of participants: Patients with diabetes mellitus (TIDM or TIIDM), aged >18 years. The studies should be specifically focused on the foot and ankle.
- Types of studies: Psychometric validation studies on patient-reported outcome measures, published in English or Spanish.
- Types of outcomes: Psychometric or clinimetric properties based on criteria according to Terwee (content validity, internal consistency, criterion validity, construct validity, reproducibility, agreement, reliability, responsiveness, floor/ceiling effect and interpretability) or COSMIN (structural validity, internal consistency, reliability, measurement error, hypothesis testing for construct validity, cross cultural validity/measurement invariance, criterion validity and responsiveness).

### 2.4. Exclusion Criteria

- Types of studies: Studies using questionnaires without evidence supporting their validity or reliability.

### 2.5. Quality Appraisal

The updated COSMIN checklist was used to evaluate the methodological quality of the studies performed to investigate the measurement properties of a PROM [21]. This standard can be used either to assess the methodological quality of studies of PROMs [22] or to compare the measurement properties of several such instruments in a systematic review [23]. Measurement properties are considered with respect to three domains: reliability, validity and responsiveness. Each property contains various items, evaluated on a 4-point Likert scale as poor, fair, good or excellent. The “worst score counts” approach was applied to derive a final rating for the PROM [23].

In addition, the studies were assessed in terms of Terwee’s psychometric properties [24]: content validity, internal consistency, criterion validity, construct validity, reproducibility (agreement and reliability), responsiveness, floor/ceiling effects and interpretability. Each issue was rated as positive “+” (adequate description or value or measure or argument related to psychometric property), negative “-” (inadequate or values under the accepted standards in each psychometric property), indeterminate “?” (doubtful methods or measures or design) or absent “0” (no information available about a psychometric property), except for responsiveness, which was rated only as present/absent.

### 2.6. Study Selection

Two blinded reviewers (XXX) (XXX) evaluated the search results, and all the reference lists were independently reviewed to ensure that the inclusion criteria were met. Disagreements were resolved by discussion between the two evaluators, or if consensus was not possible, further opinion was sought (XXXX) (XXX).

### 2.7. Data Extraction

The following data were extracted from each study using a standardised template: full title, country, year of publication; dimensions and number of items; population used for the validation process; psychometric properties by Terwee’s criteria with a positive rating; cross-cultural adaptation into different languages of each questionnaire; methodological quality according to COSMIN.

## 3. Results

**Figure 1:**
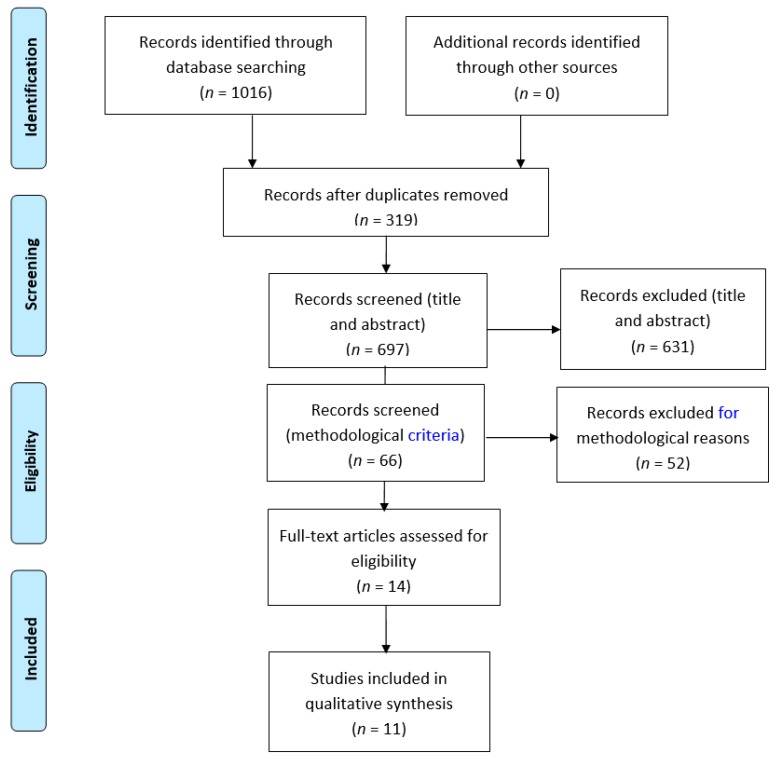
PRISMA flow diagram [19]. For more information, visit www.prisma-statement.org.

A potential 1016 studies were identified, but of these 319 were duplicates across the different databases. The remaining 697 were screened against the inclusion/exclusion criteria, using the titles, abstracts and keywords. This process led to 631 studies being discarded, in most cases because they were not psychometric validation studies of patient-reported outcomes or because they were not focused on the foot and ankle. Application of the quality appraisal filter led to the exclusion of a further 52 studies. After a detailed reading of the remaining 14 papers, three were excluded, and 11 were judged appropriate for the final qualitative review. Figure 1 shows the PRISMA flow diagram for the studies included in this review. The characteristics of each paper are summarised in Table 1, Table 2, Table 3, Table 4 and Table 5.

### 3.1. Population

The 11 studies considered included a total of 2007 participants, of whom 45.88% were male and 43.2% female, with a mean age of 61 years. Most of the participants had TIIDM (insulin-dependence).

### 3.2. Dimensions and Items

The PROMs included in the papers finally reviewed were fairly homogeneous with respect to the number of items and dimensions. The latter ranged from one in the Foot Self-Care Behavior Scale (FCBS) [58] to eight in the Foot Health Status Questionnaire (FHSQ) [5].

The areas addressed in the studies included self-care (diet, blood glucose, self-monitoring), pain, perceived health status and quality of life (quality of life, general foot health or foot health) or disability (activities of daily living, disability, limitation of function, activity restriction or sport and recreational function).

With respect to the number of items included, the PROMs ranged from long versions, with 29, for the Diabetic Foot Ulcer Scale-Short Form (DFS-SF) [29], to a mere seven items, in the Foot Self-Care Behavior Scale (FSCB) [58].

The most commonly used PROMs were the Cardiff Wound Impact Schedule (CWIS) [33] and the Foot and Ankle Ability Measures (FAAM) [43], which were similar in terms of dimensions and items, with three and two dimensions, and 26 and 29 items, respectively.

### 3.3. Psychometric Properties

The psychometric properties considered, in accordance with the Terwee criteria for each PROM, are summarised in Table 2 and Table 3.

#### 3.3.1. Content Validity

In all cases, the PROMs gave a clear description of the measurement aim and the target population and defined the criteria for item selection and exclusion. In addition, some (CWIS, DFS-SF, DFSQ-UMA) detailed the interpretability of the items, although this is not an essential characteristic for content validity.

#### 3.3.2. Internal Consistency

Internal consistency was evaluated by Cronbach’s alpha, either for the entire instrument or for each sub-scale. Seven PROMs (NeuroQol, DFS-SF, DHPSC, FCBS, DFSQ-UMA, HRQLQDFU and FHSQ) obtained a positive rating, with values ranging from 0.7 to 0.95. Two (CWIS and AOFAS-DFQ) had a negative rating, with values >0.95. No information was available in this respect for FAAM and Q-DFD, and so they were both rated zero.

#### 3.3.3. Criterion Validity

None of the PROMs obtained a positive rating for this property, which required a strong correlation with the gold standard ≥0.7. Most of the PROMs (NeuroQol, DFS-SF, CWIS, FAAM, DHPSC FCBS and DFSQ-UMA) had a negative rating, with only weak correlation. Q-DFD, HRQLQDFU and FHSQ provided no information regarding the gold standard, and AOFAS-DFQ was deficient in its methodology in comparison with the gold standard.

#### 3.3.4. Construct Validity

In this respect, none of the PROMs were rated positively. Either specific hypotheses were not formulated or less than 75% of the results obtained were in accordance with the study hypotheses, or this criterion was absent.

#### 3.3.5. Reproducibility

*Agreement*: None of the PROMs had a positive rating for measurement error, either because the minimally important change (MIC) was not defined or because they did not refer exactly to the values. In most cases (AOFAS-DFQ, FAAM, DHPSC, FCBS, HRQLQDFU and FHSQ), the rating was 0 (no information available).

*Reliability*: AOFAS-DFQ, DHPSC, FCBS, DFSQ-UMA and FHSQ obtained a positive value for this property, with an intraclass correlation coefficient (ICC) greater than 0.7. The remaining PROMs presented lower values (ICC < 0.7), or were deficient in their design or provided no information in this respect.

#### 3.3.6. Responsiveness

In this category, most of the PROMs (NeuroQol, FAAM, Q-DFD, DHPSC, DFSQ-UMA, HRQLQDFU and FHSA) had a value of 0, as they provided no information on the Smallest Detectable Change (SDC). The remaining measures did address this question, but either the methodology applied was doubtful or no evidence was provided of a clinically important change.

#### 3.3.7. Floor/Ceiling Effect

Floor/ceiling effects were only described for DHPSC (4.59%). Another seven PROMs (DFS-SF, CWIS, FAAM, Q-DFD, FCBS, HRQLQDFU and FHSQ) provided no information in this respect. AOFAS-DFQ and DFSQ-UMA aroused doubts concerning the study design employed, while in NeuroQol the floor effect was only slight and there was little evidence of a ceiling effect.

#### 3.3.8. Interpretability

Most of the PROMs considered failed to define MIC and were classed as ‘Indeterminate’.

### 3.4. Cross-Cultural Adaptation

In this respect, the PROMs varied widely, ranging from those providing no adaptation at all (DHPSC, DFSQ-UMA and HRQLQDFU) to the FAAM instrument, which has been adapted into 11 different languages (Brazilian, Chinese, Dutch, French, German, Italian, Japanese, Persian, Thai, Turkish and Spanish).

### 3.5. Methodological Quality

FHSQ obtained the best results in terms of methodological quality, according to the COSMIN criteria, see Table 4. This instrument scored positively for internal consistency, reliability, hypothesis testing for construct validity, cross-cultural validity and responsiveness, indeterminate values in measurement error and criterion validity. The only negative value recorded was for structural validity.

The next-best-performing instruments in this regard were DHPSC and FCBS, which obtained positive values for four criteria.

#### 3.5.1. Structural Validity

None of the PROMs obtained a positive value for this property. Most of them (NeuroQol, DFS-SF, CWIS, AOFAS-DFQ, DHPSC and DFSQ-UMA) were classed as ‘Indeterminate’, while FAAM, Q-DFD, FCBS, HRQLQDFU and FHSQ provided no information in this respect and were given a negative rating.

#### 3.5.2. Internal Consistency

All of the PROMs except AOFAS-DFQ, FAAM and Q-DFD obtained a positive rating for internal consistency. The Cronbach’s alpha was ≥0.70 for each subscale.

#### 3.5.3. Reliability

For reliability, AOFAS-DFQ, DHPSC, FDBS, DFSQ-UMA and FHSQ obtained ICC ≥ 0.70. The remaining PROMs were considered ‘Indeterminate’, with the exception of DFS-SF and Q-DFD, which provided no evidence in this regard and were rated negatively.

#### 3.5.4. Measurement Error

In no case was the minimal important change (MIC) defined, and so all of the PROMs were classed as ‘Indeterminate’ for measurement error.

#### 3.5.5. Hypothesis Testing for Construct Validity

For most of the PROMs (NeuroQol, DFS-SF, CWIS, FAAM, DHPSC, FCBS and FHSQ) a study hypothesis was defined and it was corroborated by the results obtained. Therefore, the instrument received a positive score.

#### 3.5.6. Cross-Cultural Validity/Measurement Invariance

Only three PROMs (AIFAS-DFQ, FAAM and FHSQ) obtained a positive score for this property. Of the rest, DFS-SF, CWIS, Q-DFD, DHPSC, HRQLQDFU were scored as indeterminate and NeuroQol, FCBS and DFSQ-UMA were rated negatively because no important differences were found between group factors or by differential item functioning.

#### 3.5.7. Criterion Validity

None of the PROMs scored positively in this respect, and most were given a negative rating due to lack of information or poor correlation.

#### 3.5.8. Responsiveness

Seven PROMs were positively rated for responsiveness because the results obtained were consistent with the study hypothesis. Only AOFAS-DFQ, Q-DFD, DFSQ-UMA and HRQLQDFU scored negatively, with study results that were not in accordance with the hypothesis.

### 3.6. Methodological Quality Scores Per Study on A Measurement Property

The methodological quality scores obtained are summarised in Table 5. In this respect, only DHPSC, FCBS AND FHSQ obtained more positive than negative values and were eligible for evaluation. However, analysis of the methodological quality scores per study on a measurement property showed that none were of excellent quality; indeed, in most cases, this quality was very low.

The overall level of quality of the PROMs considered was low. FCBS obtained the best score, with an excellent rating for internal consistency and content validity, a good rating for reliability, a fair rating for hypothesis testing and a poor rating for measurement error, structural validity, criterion validity and responsiveness. None of the PROMs were evaluated for cross-cultural validity as the inclusion criteria applied limited the studies to the context of DM.

## 4. Discussion

The aim of this systematic review was to identify PROMs used to measure the effects of DM on the foot and ankle and to evaluate the methodological quality and psychometric properties of these PROMs.

Our literature search identified only 11 PROMs aimed at patients with DM, with reference to foot and ankle pathologies. Of these instruments, the Foot Health Status Questionnaire (FHSQ) provided the best overall psychometric properties, based on COSMIN, obtaining positive values for five properties: internal consistency, reliability, hypothesis test for construct validity, cross-cultural validity and responsiveness. The only negative value obtained was for structural validity, about which no information was provided, while measurement error and criterion validity were classed as indeterminate due to a lack of information regarding MIC and correlation with the gold standard, respectively.

The FHSQ is intended to be self-administered and was initially developed and validated to evaluate the effectiveness of surgical and conservative treatment for pathologies such as skin, nail, neurological, orthopaedic and musculoskeletal disorders [62,63,64]. This PROM has more dimensions than the others considered, examining the following eight areas: foot pain, foot function, footwear, general foot health, general health, social capacity, physical activity and vigour. However, in terms of applicability, it is merely average, with a total of 17 items. Each domain comprises a question-specific number, with four questions considering pain, four regarding function, three on footwear and two on general foot health. The possible scores range from 0 to 100, representing the worst and best states, respectively, of foot health imaginable.

Only two transcultural adaptations have been made of this questionnaire, into Brazilian-Portuguese [60] and Spanish [61]. In the first case, this adaptation was implemented with a population suffering from rheumatoid arthritis. The Spanish-language version was initially used with a healthy population and later adapted to evaluate the alterations to the quality of life and foot health among patients with type I or II DM [5].

The FAAM is the most commonly used PROM for foot and ankle pathologies, being available in 11 different languages, although not all of these adaptations are specifically intended for patients with DM. However, at the methodological level, this instrument presented positive values only for hypothesis testing for construct validity, cross-cultural validity and responsiveness. This finding suggests that very careful preparation is needed before performing transcultural adaptation into other languages or with respect to specific pathologies.

In line with the study goals, in this review, we identify and evaluate PROMs designed for patients with DM, with particular respect to foot and ankle pathologies. The methodological quality of each PROM is assessed. We observe that although reviews have been conducted previously on the impact of DM on the foot and ankle [16], sometimes narrowly focused on the rheumatoid foot [65], while others address the question more broadly [66] or are related to pain or dysfunction in particular [67], in every case they are deficient in the sense that appropriate methodological guidelines are not followed. In our opinion, the most up-to-date and rigorous methodological criteria for such reviews are those proposed by COSMIN. Further investigation in this field is needed to fill the research gaps observed in the PROMs analysed in this paper, perhaps focusing first on those scoring highest in our review and taking into account the COSMIN checklist for this purpose.

The application of PROMs in clinical practice is an important issue, and especially in the pathologies we discuss, because they often include screening and monitoring functions, as a means of promoting patient-centered care, as a decision-making aid, in order to facilitate communication amongst multidisciplinary teams and to monitor the quality of patient care [68]. Evidence suggests that the use of PROMs in clinical practice helps detect HRQoL problems but has less impact on how clinicians manage patient problems or on subsequent patient outcomes. Despite the deficiencies observed, at present, the PROMs considered are the only instruments currently available for identifying and evaluating foot and ankle pathologies in all patients, irrespective of their geographic location.

## 5. Limitations

This study presents significant limitations. Firstly, very few PROMs have been designed for patients with DM, with particular respect to foot and ankle pathologies. Furthermore, many of the instruments analysed in our review lack important information in many respects: some fail to describe the type of diabetes, others do not report the proportions of patients with and without DM, while others present a statistical analysis that is not corroborated by the necessary data. Information in this respect was requested from the respective authors, but no response was obtained.

## 6. Clinical Implications

The present review offers useful information to researchers and clinicians regarding the PROMs that have been proposed for patients with DM and with foot and ankle pathologies. A detailed analysis is made of the methodological quality of each such PROM.

## 7. Conclusions

Noting the low overall methodological quality of the PROMs considered, with respect to foot and ankle pathologies in patients with DM, we conclude that the most appropriate questionnaire currently available is the Foot Health Status Questionnaire for diabetic patients.

## Figures and Tables

**Table 1:** Instrument included.

Acronym	Full Title	Author	Country
NeuroQol	Neuropathy Quality of Life Instrument	Loretta Vileikyte et al.	UK and USA
DFS-SF	Diabetic Foot Ulcer Scale–Short Form	Carla M. Bann et al.	USA
CWIS	Cardiff Wound Impact Schedule	Patricia Price and Keith Harding	UK
AOFAS-DFQ	American Orthopaedic Foot and Ankle Society	Vibhu Dhawan et al.	USA
FAAM	Foot and Ankle Ability Measure	RobRoy L. Martin et al.	USA
Q-DFD	Questionnaire for Diabetes Related Foot Disease	Shan M Bergin et al.	Australia
DHPSC	Diabetes Health Promotion Self-Care Scale	Ruey-Hsia Wang et al.	Taiwan
FCBS	Foot Self-Care Behavior Scale	Yen-Fan Chin and Tzu-Ting Huang	Taiwan
DFSQ-UMA	Diabetic Foot Self-care Questionnaire of University of Malaga	Emmanuel Navarro et al.	Spain
HRQLQDFU	Health Related Quality of Life Questionnaire in Diabetic Foot Ulcer Patients	Ramya Kateel et al.	India
FHSQ	Foot Health Status Questionnaire	Patricia Palomo-López et al.	Spain

**Table 2:** Characteristic of all instruments.

	Year	Dimensions and Number of Items	Population Used for Validation	Psychometric Properties	Cross-Cultural Adaptation
NeuroQoL [25]	2003	6 dimensions: painful symptoms and paresthesia, symptoms of reduced/lost feeling in the feet, diffuse sensory motor symptoms, limitations in daily activities, interpersonal problems and emotional burden 28 items	418 patients with diabetic peripheral neuropathy *n*: 296 male (70.8%) *n*: 122 female (29.2%) Mean age: 61.76 years	- Internal consistency: Cronbach’s alpha (0.86–0.95)	Spanish [26], Chinese [27], Brazilian [28]
DFS-SF [29]	2003	6 dimensions: leisure, physical health, dependence/daily life, negative emotions, worried about ulcers/feet, bothered by ulcer care 29 items	218 (diabetic with foot ulcer) and 108 (placebo) patients: *n*: 241male (74%) *n*: 85 female (26%) Age 27–87 years	- Internal consistency: Cronbach’s alpha (0.74–0.94)	Greek [30], Polish [31], Chinese [32]
CWIS [33]	2004	3 dimensions: physical symptoms and daily living, social life and well-being 26 items + overall and individual rating of HRQoL	87 with leg ulceration and 48 with diabetic foot ulceration *n*: 74 male (55%) *n*: 61 female (45%) Mean age 65.9 years (43–85.5)		Canadian [34], Chinese [34], French, German, English (US) [34], Sinhala [35], Swedish [36], Brazilian [34]
AOFAS-DFQ [37]	2005	6 dimensions: general health, care, worry, sleep, emotion and physicality.	57 patients diagnosed with Charcot arthropathy *n*: 25 male (57.8%) *n*: 33 female (42.2%) Mean age: 57.5 years (37.7–80.6)	- Test-retest 0.77	Dutch [38], German [39], Italian [40], Persian [41], Turkish [42]
FAAM [43]	2009	2 dimensions: activities of daily living and sports 21 + 8 items	83 patients with diabetes and foot and ankle problems *n*: 45 male (54%) *n*: 38 female (46%) Mean age: 60.3 years (21–93)		Brazilian [44], Chinese [45], Dutch [46], French [47], German [48], Italian [49], Japanese [50], Persian [51], Thai [52], Turkish [53], Spanish [54]
Q-DFD [55]	2009	5 dimensions: peripheral neuropathy, peripheral vasculopathy, foot ulceration, amputation and foot deformity 12 items	31 patients with diabetes *n*:15 male (48%) *n*: 16 female (52%) Mean age: 64 years (45–80)		Spanish [56]
DHPSC [57]	2012	7 dimensions: interpersonal relationships, diet, blood glucose self-monitoring, personal health responsibility, exercise, adherence to the recommended regimens, and foot care 26 items	489 patients with Type II diabetes *n*: 243 male (49.7%) *n*: 246 female (50.3%) Mean age: 58.1 years	- Internal consistency: Cronbach’s alpha 0.88 - Reliability: ICC 0.94 - Floor and ceiling effects: 4.59%	
FCBS [58]	2013	1 dimension 7 items	295 patients with diabetes *n*: 151 male (51.2%) *n*: 144 female (48.8%) Mean age: 66.93 years	-Internal consistency: Cronbach’s alpha 0.73 - Reliability: test-retest 0.92	Mexican [58]
DFSQ-UMA [59]	2015	3 dimensions: self-care, foot care, and footwear and socks 16 items	209 with diabetes (48 type I and 161 type II) *n*: 101 male (48%) *n*: 108 female (52%) Mean age: 57.78 years (male) and 64.66 years (female)	- Internal consistency: Chronbach’s alpha 0.89 - Reliability: ICC 0.89–0.92	
HRQLQDFU [35]	2017	6 dimensions: physical health, daily activity, social, physical symptoms, emotional and financial 20 items	10 patients with diabetic foot ulcers *n*: 7 male (70%) *n*: 3 female (30%) Mean age: 65 years	- Internal consistency: Cronbach’s alpha 0.86	
FHSQ [5]	2018	8 dimensions: foot pain, foot function, footwear, general foot health, general health, social capacity, physical activity, and vigour 17 items	62 patients (31 type I and 31 type II) *n*:22 male (35.5%) *n*: 40 females (64.5%) Mean age: 59 years (30–86)	- Internal consistency: Cronbach’s alpha 0.89–0.95 - Reliability: ICC 0.74–0.92	Brazilian [60], Spanish [61]

N: Number of subjects; ICC: Intraclass correlation coefficient.

**Table 3:** Summary of the assessment of the measurement properties of all questionnaires.

	Content Validity	Internal Consistency	Criterion Validity	Construct Validity	Reproduci bility Agreement	Reproduci bility Reliability	Responsiveness	Floor and Ceiling Effects	Interpretability	Final Assessment
NeuroQol	+	+	-	-	?	?	0	-	?	
DFS-SF	+	+	-	?	?	-	?	0	0	
CWIS	+	-	-	-	?	?	?	0	?	
AOFAS-DFQ	+	-	?	?	0	+	?	?	?	
FAAM	+	0	-	?	0	0	0	0	?	
Q-DFD	+	0	0	?	?	-	0	0	0	
DHPSC	+	+	-	-	0	+	0	+	?	√
FCBS	+	+	-	-	0	+	?	0	?	
DFSQ-UMA	+	+	-	-	?	+	0	?	?	
HRQLQDFU	+	+	0	0	0	?	0	0	0	
FHSQ	+	+	0	?	0	+	0	0	?	

Rating: + Positive rating; ? Indeterminate rating; - Negative rating; 0 No information available.

**Table 4:** Detailed COSMIN rating.

	Structural Validity	Internal Consistency	Reliability	Measurement Error	Hypothesis Testing for Construct Validity	Cross-cultural Validity	Criterion Validity	Responsiveness
NeuroQol	?	+	?	?	+	-	-	+
DFS-SF	?	+	-	?	+	?	-	+
CWIS	?	+	?	?	+	?	-	+
AOFAS-DFQ	?	-	+	?	?	+	?	?
FAAM	-	?	?	?	+	+	-	+
Q-DFD	-	?	-	?	?	?	?	?
DHPSC	?	+	+	?	+	?	-	+
FCBS	-	+	+	?	+	-	-	+
DFSQ-UMA	?	+	+	?	?	-	-	?
HRQLQDFU	-	+	?	?	?	?	?	?
FHSQ	-	+	+	?	+	+	?	+

Rating: “+”: Positive rating; “?”: Indeterminate rating; “-“: Negative rating.

**Table 5:** Methodological quality scores per patient-reported outcome measures (PROMs) on a measurement property.

	BOX AInternal Consistency	BOX BReliability	BOX CMeasurement Error	BOX DContent Validity	BOX EStructural Validity	BOX FHypothesis Testing	BOX GCross-cultural Validity	BOX HCriterion Validity	BOX IResponsiveness
DHPSC	EXCELLENT	POOR	POOR	EXCELLENT	POOR	FAIR	-	POOR	POOR
FCBS	EXCELLENT	GOOD	POOR	EXCELLENT	POOR	FAIR	-	POOR	POOR
FHSQ	FAIR	POOR	POOR	POOR	POOR	POOR	-	POOR	POOR

## Supplementary Materials

The following are available online at http://www.mdpi.com/2077-0383/8/2/146/s1, Searching strategy.
