# Supplementary material for: Patient-Reported Outcome Measures for Patients with Diabetes Mellitus Associated with Foot and Ankle Pathologies: A Systematic Review
Source: J Clin Med. 2019 Jan 27;8(2):146. doi: 10.3390/jcm8020146 (PMC6407033; doi:10.3390/jcm8020146)
Supplement: Supplementary file 1 [file jcm-08-00146-s001.pdf]

Apendix A. Searching strategy.

(diabet[tiab] OR diabeta[tiab] OR diabeta'[tiab] OR diabetacorum[tiab] OR diabetaction[tiab] OR diabetaeta[tiab] OR diabetagenic[tiab] OR diabetas[tiab] OR diabetc[tiab] OR diabetcieza[tiab] OR diabetcs[tiab] OR diabete[tiab] OR diabete's[tiab] OR diabetea[tiab] OR diabetec[tiab] OR diabeted[tiab] OR diabeteic[tiab] OR diabeteics[tiab] OR diabetelogical[tiab] OR diabetelogy[tiab] OR diabeterelated[tiab] OR diabetergestemd[tiab] OR diabeters[tiab] OR diabetes[tiab] OR diabetes'[tiab] OR diabetes''[tiab] OR diabetes'african[tiab] OR diabetes'data[tiab] OR diabetes'risk[tiab] OR diabetes's[tiab] OR diabetes1[tiab] OR diabetes11[tiab] OR diabetes2[tiab] OR diabetes3[tiab] OR diabetes39[tiab] OR diabetesa[tiab] OR diabetesassociated[tiab] OR diabetesatlas[tiab] OR diabetesbaicalin[tiab] OR diabetesbb[tiab] OR diabetesbut[tiab] OR diabetescare[tiab] OR diabetescarvone[tiab] OR diabetescoach[tiab] OR diabetescpme[tiab] OR diabetesde[tiab] OR diabetesdriving[tiab] OR diabetesdue[tiab] OR diabetese[tiab] OR diabeteses[tiab] OR diabetesexercise[tiab] OR diabetesexercised[tiab] OR diabetesforeningen[tiab] OR diabetesforms[tiab] OR diabetesgenes[tiab] OR diabetesii[tiab] OR diabetesin[tiab] OR diabetesincidencia[tiab] OR diabetesinduced[tiab] OR diabetesinsipidus[tiab] OR diabetesinsulin[tiab] OR diabetesis[tiab] OR diabeteskockazat[tiab] OR diabetesl[tiab] OR diabeteslike[tiab] OR diabeteslive[tiab] OR diabetesmeds[tiab] OR diabetesmellitus[tiab] OR diabetesmine[tiab] OR diabetesmortalitas[tiab] OR diabetesoutcomequality[tiab] OR diabetespatienten[tiab] OR diabetesplanning[tiab] OR diabetespresenting[tiab] OR diabetesprevalencia[tiab] OR diabetespro[tiab] OR diabetesproduced[tiab] OR diabetesprone[tiab] OR diabetesrask[tiab] OR diabetesrelated[tiab] OR diabetesrisks[tiab] OR diabetesrobert[tiab] OR diabetesscenariosforjuniordoctors[tiab] OR diabetessoftware[tiab] OR diabetesspecific[tiab] OR diabetesstation[tiab] OR diabetesstudy[tiab] OR diabetessystematic[tiab] OR diabetesthe[tiab] OR diabetestherapie[tiab] OR diabetesthere[tiab] OR diabetestrial[tiab] OR diabetesvereniging[tiab] OR diabetesweekly[tiab] OR diabetesxglucose[tiab] OR diabeteszentrum[tiab] OR diabetetes[tiab] OR diabetetic[tiab] OR diabetetogenesis[tiab] OR diabetetogenic[tiab] OR diabetets[tiab] OR diabetex[tiab] OR diabetgic[tiab] OR diabethic[tiab] OR diabeti[tiab] OR diabetia[tiab] OR diabetic[tiab] OR diabetic'[tiab] OR diabetic''[tiab] OR diabetic's[tiab] OR diabetica[tiab] OR diabetica'[tiab] OR diabetical[tiab] OR diabetically[tiab] OR diabeticand[tiab] OR diabeticas[tiab] OR diabeticassociated[tiab] OR diabeticcomplications[tiab] OR diabeticdyslipidemia[tiab] OR diabetice[tiab] OR diabeticfoot[tiab] OR diabeticfoveal[tiab] OR diabetichip[tiab] OR diabetichip's[tiab] OR diabetici[tiab] OR diabeticians[tiab] OR diabeticin[tiab] OR diabeticke[tiab] OR diabeticketoacidosis[tiab] OR diabeticlike[tiab] OR diabeticmen[tiab] OR diabeticmothers[tiab] OR diabeticnephropathy[tiab] OR diabetico[tiab] OR diabeticorum[tiab] OR diabeticos[tiab] OR diabeticpatients[tiab] OR diabeticpolyneuropathy[tiab] OR diabeticrats[tiab] OR diabeticretinopathy[tiab] OR diabetics[tiab] OR diabetics'[tiab] OR diabetics's[tiab] OR diabetics1[tiab] OR diabeticses[tiab] OR diabeticsts[tiab] OR diabetictype[tiab] OR diabeticum[tiab] OR diabeticus[tiab] OR diabetid[tiab] OR diabetiee[tiab] OR diabeties[tiab] OR diabetietes[tiab] OR diabetiform[tiab] OR diabetigenesis[tiab] OR diabetiker[tiab] OR diabetikerbund[tiab] OR diabetikerkost[tiab] OR diabetikern[tiab] OR diabetimss[tiab] OR diabetin[tiab] OR diabetinol[tiab] OR diabetio[tiab] OR diabetiogenic[tiab] OR

diabetiologists[tiab] OR diabetique[tiab] OR diabetique'[tiab] OR diabetiques[tiab] OR diabetis[tiab] OR diabetische[tiab] OR diabetisches[tiab] OR diabetised[tiab] OR diabetisity[tiab] OR diabetisk[tiab] OR diabetiva[tiab] OR diabetization[tiab] OR diabetized[tiab] OR diabetlic[tiab] OR diabetlmss[tiab] OR diabetmin[tiab] OR diabeto[tiab] OR diabetobiguanides[tiab] OR diabetogen[tiab] OR diabetogene[tiab] OR diabetogenecity[tiab] OR diabetogeneesis[tiab] OR diabetogeneic[tiab] OR diabetogeneicity[tiab] OR diabetogenensis[tiab] OR diabetogenes[tiab] OR diabetogenes'[tiab] OR diabetogenesis[tiab] OR diabetogenetic[tiab] OR diabetogenic[tiab] OR diabetogenic'[tiab] OR diabetogenicity[tiab] OR diabetogenics[tiab] OR diabetogenis[tiab] OR diabetogenous[tiab] OR diabetogens[tiab] OR diabetoid[tiab] OR diabetol[tiab] OR diabetolgist[tiab] OR diabetolgists[tiab] OR diabetologem[tiab] OR diabetologi[tiab] OR diabetologia[tiab] OR diabetologia12[tiab] OR diabetologic[tiab] OR diabetologica[tiab] OR diabetologica'[tiab] OR diabetological[tiab] OR diabetologically[tiab] OR diabetologico[tiab] OR diabetologie[tiab] OR diabetologiques[tiab] OR diabetologist[tiab] OR diabetologist's[tiab] OR diabetologists[tiab] OR diabetologists'[tiab] OR diabetologits[tiab] OR diabetologue[tiab] OR diabetology[tiab] OR diabetology'[tiab] OR diabetologyteam[tiab] OR diabetolytic[tiab] OR diabetomobil[tiab] OR diabetomobile[tiab] OR diabeton[tiab] OR diabetoneuropathic[tiab] OR diabetoporosis[tiab] OR diabetor[tiab] OR diabetorenal[tiab] OR diabetorum[tiab] OR diabetosan[tiab] OR diabetotherapy[tiab] OR diabetric[tiab] OR diabetricorum[tiab] OR diabets[tiab] OR diabetss[tiab] OR diabetter[tiab] OR diabettics[tiab] OR diabetus[tiab])

AND

(patient-reported-outcomes[tiab] OR instrument[tiab] OR questionnaire[tiab] OR index[tiab] OR inventory[tiab] OR scale[tiab] OR (psychometrc[tiab] OR psychometri[tiab] OR psychometric[tiab] OR psychometric'[tiab] OR psychometrica[tiab] OR psychometrical[tiab] OR psychometrically[tiab] OR psychometrically'[tiab] OR psychometricallydefined[tiab] OR psychometrican[tiab] OR psychometricevaluation[tiab] OR psychometrician[tiab] OR psychometricians[tiab] OR psychometricians'[tiab] OR psychometricly[tiab] OR psychometricproperties[tiab] OR psychometrics[tiab] OR psychometrics'[tiab] OR psychometrie[tiab] OR psychometries[tiab] OR psychometrika[tiab] OR psychometrika's[tiab] OR psychometrique[tiab] OR psychometriques[tiab] OR psychometris[tiab] OR psychometrische[tiab] OR psychometrist[tiab] OR psychometrists[tiab] OR psychometry[tiab] OR psychometry's[tiab]) OR (reliab[tiab] OR reliabaly[tiab] OR reliabe[tiab] OR reliabel[tiab] OR reliabely[tiab] OR reliabiities[tiab] OR reliabiity[tiab] OR reliabil[tiab] OR reliabile[tiab] OR reliabiligy[tiab] OR reliabiliity[tiab] OR reliabililty[tiab] OR reliabilism[tiab] OR reliabilist[tiab] OR reliabilists[tiab] OR reliabilit[tiab] OR reliabilitat[tiab] OR reliabilites[tiab] OR reliabilities[tiab] OR reliabilitiy[tiab] OR reliability[tiab] OR reliability'[tiab] OR reliability's[tiab] OR reliability0[tiab] OR reliabilityand[tiab] OR reliabilityat[tiab] OR reliabilityobjective[tiab] OR reliabilityof[tiab] OR reliabiliy[tiab] OR reliabillty[tiab] OR reliabiltiy[tiab] OR reliabilty[tiab] OR reliabily[tiab] OR reliabity[tiab] OR reliabl[tiab] OR reliable[tiab] OR reliable'[tiab] OR reliableanalytical[tiab] OR reliabledata[tiab] OR reliableduring[tiab] OR reliablefertility[tiab] OR reliablefor[tiab] OR reliablegenome[tiab] OR reliablemeans[tiab] OR reliableness[tiab] OR

reliablenesses[tiab] OR reliableoutcome[tiab] OR reliableover[tiab] OR reliablereporters[tiab] OR reliables[tiab] OR reliablethat[tiab] OR reliablethe[tiab] OR reliablility[tiab] OR reliablitity[tiab] OR reliablity[tiab] OR reliablity'[tiab] OR reliably[tiab] OR reliablydistinguish[tiab] OR reliaby[tiab]) OR alpha[tiab])

AND

(("bones of lower extremity"[MeSH Terms] OR ("bones"[All Fields] AND "lower"[All Fields] AND "extremity"[All Fields]) OR "bones of lower extremity"[All Fields]) OR ("lower extremity"[MeSH Terms] OR ("lower"[All Fields] AND "extremity"[All Fields]) OR "lower extremity"[All Fields]) OR ("foot"[MeSH Terms] OR "foot"[All Fields]) OR ("foot"[MeSH Terms] OR "foot"[All Fields] OR "feet"[All Fields]) OR ("ankle"[MeSH Terms] OR "ankle"[All Fields] OR "ankle joint"[MeSH Terms] OR ("ankle"[All Fields] AND "joint"[All Fields]) OR "ankle joint"[All Fields]) OR ("ankle joint"[MeSH Terms] OR ("ankle"[All Fields] AND "joint"[All Fields]) OR "ankle joint"[All Fields]) OR ("subtalar joint"[MeSH Terms] OR ("subtalar"[All Fields] AND "joint"[All Fields]) OR "subtalar joint"[All Fields]))

AND

(("pain"[MeSH Terms] OR "pain"[All Fields]) OR disability[All Fields] OR (funct[All Fields] OR functaional[All Fields] OR functal[All Fields] OR functed[All Fields] OR functflr[All Fields] OR functhon[All Fields] OR functi[All Fields] OR functi'os[All Fields] OR functia[All Fields] OR functial[All Fields] OR functiaonalization[All Fields] OR functiaonalized[All Fields] OR functie[All Fields] OR functiebehoud[All Fields] OR functiebepaling[All Fields] OR functiebeperking[All Fields] OR functiediagnostiek[All Fields] OR functiedifferentiatie[All Fields] OR functiegericht[All Fields] OR functieherstel[All Fields] OR functiei[All Fields] OR functieleen[All Fields] OR functieleer[All Fields] OR functieleet[All Fields] OR functieler[All Fields] OR functieloze[All Fields] OR functieonderzoek[All Fields] OR functieproef[All Fields] OR functieproeven[All Fields] OR functieprofiel[All Fields] OR functies[All Fields] OR functiestoornis[All Fields] OR functiestoornissen[All Fields] OR functietesten[All Fields] OR functieverandering[All Fields] OR functieverbeterin[All Fields] OR functieverlies[All Fields] OR functiewaardering[All Fields] OR functii[All Fields] OR functiile[All Fields] OR functiilor[All Fields] OR functiion[All Fields] OR functiional[All Fields] OR functille[All Fields] OR functimal[All Fields] OR functin[All Fields] OR functinal[All Fields] OR functinale[All Fields] OR functinalised[All Fields] OR functinalization[All Fields] OR functinalized[All Fields] OR functing[All Fields] OR functinin[All Fields] OR functining[All Fields] OR functino[All Fields] OR functinoalized[All Fields] OR functinonal[All Fields] OR functinoning[All Fields] OR functins[All Fields] OR functio[All Fields] OR functioal[All Fields] OR functioanl[All Fields] OR functioanlization[All Fields] OR functioanlly[All Fields] OR functiobal[All Fields] OR functiof[All Fields] OR functiogenesis[All Fields] OR functiogenetic[All Fields] OR functiogenic[All Fields] OR functiogenic'[All Fields] OR functiogram[All Fields] OR functiograph[All Fields] OR functiograph'[All Fields] OR functiographs[All Fields] OR functioin[All Fields] OR functioining[All Fields] OR functioion[All Fields] OR functioja[All Fields] OR functiojanak[All Fields] OR functiojaval[All Fields] OR functiok[All Fields] OR functiol[All Fields] OR functiom[All Fields] OR function[All

Fields] OR function'[All Fields] OR function"[All Fields] OR function'alis[All Fields] OR function'in[All Fields] OR function's[All Fields] OR function'were[All Fields] OR function,[All Fields] OR function1[All Fields] OR function2[All Fields] OR function24[All Fields] OR function26[All Fields] OR function28[All Fields] OR function2a[All Fields] OR function2gene[All Fields] OR function3[All Fields] OR function490[All Fields] OR function647[All Fields] OR functiona[All Fields] OR functionaal[All Fields] OR functionabilities[All Fields] OR functionability[All Fields] OR functionable[All Fields] OR functionacondition[All Fields] OR functionaction[All Fields] OR functionactivities[All Fields] OR functionadded[All Fields] OR functionae[All Fields] OR functionai[All Fields] OR functionaing[All Fields] OR functionaires[All Fields] OR functionais[All Fields] OR functionaities[All Fields] OR functionakimageanalysis[All Fields] OR functional[All Fields] OR functional'[All Fields] OR functional"[All Fields] OR functional'noe[All Fields] OR functional'proximal[All Fields] OR functional's[All Fields] OR functionala[All Fields] OR functionalactivity[All Fields] OR functionalal[All Fields] OR functionalanalysis[All Fields] OR functionalanalytical[All Fields] OR functionaland[All Fields] OR functionalbeta[All Fields] OR functionalbiological[All Fields] OR functionalclass[All Fields] OR functionalconstipation[All Fields] OR functionaldarsallele[All Fields] OR functionaldiagnostic[All Fields] OR functionale[All Fields] OR functionalefficiency[All Fields] OR functionalenzyme[All Fields] OR functionales[All Fields] OR functionalflow[All Fields] OR functionalgastroenterology[All Fields] OR functionalgenomics[All Fields] OR functionalglycomics[All Fields] OR functionalgrazers[All Fields] OR functionalgroup[All Fields] OR functionalgroupentity[All Fields] OR functionalgroups[All Fields] OR functionali[All Fields] OR functionaliation[All Fields] OR functionalied[All Fields] OR functionalimaging[All Fields] OR functionalimpairment[All Fields] OR functionalin[All Fields] OR functionalinsts[All Fields] OR functionalis[All Fields] OR functionalisability[All Fields] OR functionalisable[All Fields] OR functionalisation[All Fields] OR functionalisations[All Fields] OR functionalisative[All Fields] OR functionalise[All Fields] OR functionalised[All Fields] OR functionalised'[All Fields] OR functionalises[All Fields] OR functionalising[All Fields] OR functionalism[All Fields] OR functionalismo[All Fields] OR functionalisms[All Fields] OR functionalist[All Fields] OR functionalist'[All Fields] OR functionalist's[All Fields] OR functionalistic[All Fields] OR functionalistion[All Fields] OR functionalists[All Fields] OR functionalitate[All Fields] OR functionalitatea[All Fields] OR functionalitatii[All Fields] OR functionalitation[All Fields] OR functionalites[All Fields] OR functionalities[All Fields] OR functionalities'[All Fields] OR functionalitites[All Fields] OR functionality[All Fields] OR functionality'[All Fields] OR functionality's[All Fields] OR functionalizability[All Fields] OR functionalizable[All Fields] OR functionalizaed[All Fields] OR functionalizarea[All Fields] OR functionalizated[All Fields] OR functionalizating[All Fields] OR functionalization[All Fields] OR functionalization'[All Fields] OR functionalization's[All Fields] OR functionalization5[All Fields] OR functionalizationed[All Fields] OR functionalizationof[All Fields] OR functionalizations[All Fields] OR functionalizatlon[All Fields] OR functionalizaton[All Fields] OR functionalizd[All Fields] OR functionalize[All Fields] OR functionalizeable[All Fields] OR functionalized[All Fields] OR functionalizedwith[All Fields] OR functionalizer[All

Fields] OR functionalizers[All Fields] OR functionalizes[All Fields] OR functionalizes'[All Fields] OR functionaliziation[All Fields] OR functionalizied[All Fields] OR functionalizing[All Fields] OR functionalizion[All Fields] OR functionaliztion[All Fields] OR functionallimitations[All Fields] OR functionallities[All Fields] OR functionallized[All Fields] OR functionallly[All Fields] OR functionally[All Fields] OR functionally'[All Fields] OR functionallydependent[All Fields] OR functionallyidentical[All Fields] OR functionallywise[All Fields] OR functionalmedicine[All Fields] OR functionalmitral[All Fields] OR functionalmolecular[All Fields] OR functionalmorphology[All Fields] OR functionalmri[All Fields] OR functionalnature[All Fields] OR functionalnet[All Fields] OR functionalnoda[All Fields] OR functionaloutcomes[All Fields] OR functionalpolarities[All Fields] OR functionalpolymer[All Fields] OR functionalpost[All Fields] OR functionalproteins[All Fields] OR functionalroles[All Fields] OR functionals[All Fields] OR functionals'[All Fields] OR functionalsimilarity[All Fields] OR functionalskill[All Fields] OR functionalskills[All Fields] OR functionalsolutions[All Fields] OR functionalstarter[All Fields] OR functionalstructure[All Fields] OR functionalstudies[All Fields] OR functionalties[All Fields] OR functionalvoice[All Fields] OR functionalvs[All Fields] OR functionaly[All Fields] OR functionalysed[All Fields] OR functionalyzed[All Fields] OR functionalzation[All Fields] OR functionalzed[All Fields] OR functionalziation[All Fields] OR functionalzied[All Fields] OR functionamento[All Fields] OR functionamiento[All Fields] OR functionanalyzer[All Fields] OR functionand[All Fields] OR functionanlly[All Fields] OR functionannotator[All Fields] OR functionante[All Fields] OR functionantes[All Fields] OR functionapcpr4[All Fields] OR functionare[All Fields] OR functionarea[All Fields] OR functionaresse[All Fields] OR functionaries[All Fields] OR functionaries'[All Fields] OR functionarii[All Fields] OR functionarios[All Fields] OR functionaris[All Fields] OR functionarissen[All Fields] OR functionarization[All Fields] OR functionary[All Fields] OR functionary'[All Fields] OR functionas[All Fields] OR functionassociated[All Fields] OR functionate[All Fields] OR functionates[All Fields] OR functionating[All Fields] OR functionation[All Fields] OR functionator[All Fields] OR functionbfat[All Fields] OR functionbody[All Fields] OR functionbone[All Fields] OR functionbrix[All Fields] OR functionby[All Fields] OR functionc[All Fields] OR functioncdots[All Fields] OR functiond[All Fields] OR functione[All Fields] OR functioneal[All Fields] OR functioned[All Fields] OR functioneel[All Fields] OR functioneelanatomisch[All Fields] OR functioneert[All Fields] OR functionel[All Fields] OR functionele[All Fields] OR functionelisa[All Fields] OR functionelle[All Fields] OR functionellen[All Fields] OR functioneller[All Fields] OR functionelles[All Fields] OR functionellipsis[All Fields] OR functionellipsis'[All Fields] OR functionem[All Fields] OR functionen[All Fields] OR functioner[All Fields] OR functioneren[All Fields] OR functionerend[All Fields] OR functionerende[All Fields] OR functionerings[All Fields] OR functiones[All Fields] OR functionfor[All Fields] OR functionfunctions[All Fields] OR functiong[All Fields] OR functionh[All Fields] OR functionhalf[All Fields] OR functionhave[All Fields] OR functioni[All Fields] OR functionialized[All Fields] OR functionialy[All Fields] OR functionig[All Fields] OR functionilized[All Fields] OR functionimg[All Fields] OR functionin[All Fields] OR functioninfinity[All Fields] OR functioning[All Fields] OR functioning'[All Fields] OR functioning''[All Fields] OR functioning's[All Fields] OR

functioninga[All Fields] OR functioningand[All Fields] OR functioningfeatures[All Fields] OR functioningin[All Fields] OR functioningphysical[All Fields] OR functionings[All Fields] OR functionings'[All Fields] OR functioningt[All Fields] OR functioningthe[All Fields] OR functionioning[All Fields] OR functionisable[All Fields] OR functionization[All Fields] OR functionized[All Fields] OR functionl[All Fields] OR functionla[All Fields] OR functionlaized[All Fields] OR functionless[All Fields] OR functionless'[All Fields] OR functionlessness[All Fields] OR functionlike[All Fields] OR functionlised[All Fields] OR functionlization[All Fields] OR functionlize[All Fields] OR functionlized[All Fields] OR functionllized[All Fields] OR functionlmnagene[All Fields] OR functionly[All Fields] OR functionmelbourne[All Fields] OR functionment[All Fields] OR functionmuscle[All Fields] OR functionmutant[All Fields] OR functionn[All Fields] OR functionnal[All Fields] OR functionnale[All Fields] OR functionnalization[All Fields] OR functionnalized[All Fields] OR functionnally[All Fields] OR functionnaly[All Fields] OR functionned[All Fields] OR functionnel[All Fields] OR functionnele[All Fields] OR functionnelle[All Fields] OR functionnelles[All Fields] OR functionnels[All Fields] OR functionnement[All Fields] OR functionnes[All Fields] OR functionning[All Fields] OR functionnlrp3allele[All Fields] OR functionnon[All Fields] OR functiono[All Fields] OR functionof[All Fields] OR functionograph[All Fields] OR functionography[All Fields] OR functionold[All Fields] OR functionome[All Fields] OR functionome'[All Fields] OR functionomes[All Fields] OR functionometric[All Fields] OR functionomic[All Fields] OR functionomics[All Fields] OR functionomics'[All Fields] OR functionpattern[All Fields] OR functionpc[All Fields] OR functionpiso[All Fields] OR functionplasma[All Fields] OR functionplaster[All Fields] OR functionpost[All Fields] OR functionprediction[All Fields] OR functionprotective[All Fields] OR functionpsi[All Fields] OR functionrelated[All Fields] OR functionrelationship[All Fields] OR functionroi[All Fields] OR functions[All Fields] OR functions'[All Fields] OR functions's[All Fields] OR functionsa[All Fields] OR functionsal[All Fields] OR functionsalk[All Fields] OR functionsand[All Fields] OR functionsarriena[All Fields] OR functionsaving[All Fields] OR functionse[All Fields] OR functionsf[All Fields] OR functionship[All Fields] OR functionskane[All Fields] OR functionsleuphana[All Fields] OR functionsmduring[All Fields] OR functionsmeasured[All Fields] OR functionsmf[All Fields] OR functionsparing[All Fields] OR functionsrelated[All Fields] OR functionsreproduce[All Fields] OR functionsstorungen[All Fields] OR functionstate[All Fields] OR functionsthe[All Fields] OR functionsto[All Fields] OR functionsuccess[All Fields] OR functionsurg[All Fields] OR functionsviaepigenetic[All Fields] OR functionswechsel[All Fields] OR functionswechsels[All Fields] OR functionswere[All Fields] OR functionswithin[All Fields] OR functiont[All Fields] OR functiontesting[All Fields] OR functiontests[All Fields] OR functionthalassemia[All Fields] OR functionthat[All Fields] OR functionthe[All Fields] OR functionthere[All Fields] OR functiontheta[All Fields] OR functiontional[All Fields] OR functiontlu[All Fields] OR functiontrade[All Fields] OR functiontrpc[All Fields] OR functionum[All Fields] OR functionunderlies[All Fields] OR functionuniversity[All Fields] OR functionvariants[All Fields] OR functionwas[All Fields] OR functionwere[All Fields] OR functionwerestatisticallyanalyzed[All Fields] OR functionwhiles[All Fields] OR functionwise[All Fields] OR functionx[All Fields] OR functiony[All Fields] OR functionyoung[All Fields] OR functios[All Fields] OR functious[All Fields] OR functironal[All Fields] OR functiunii[All Fields] OR

functiunile[All Fields] OR functiunilor[All Fields] OR functive[All Fields] OR functlonal[All Fields] OR functn[All Fields] OR functnal[All Fields] OR functoional[All Fields] OR functome[All Fields] OR functon[All Fields] OR functonal[All Fields] OR functonality[All Fields] OR functonalized[All Fields] OR functonally[All Fields] OR functons[All Fields] OR functool[All Fields] OR functool2[All Fields] OR functools[All Fields] OR functor[All Fields] OR functorial[All Fields] OR functors[All Fields] OR functose[All Fields] OR functree[All Fields] OR functree's[All Fields] OR functronal[All Fields] OR functsii[All Fields] OR functsional'noe[All Fields] OR functsional'nogo[All Fields] OR functsional'nykh[All Fields] OR functsnp[All Fields] OR functtionala[All Fields] OR functual[All Fields] OR functuation[All Fields] OR functuations[All Fields] OR functuion[All Fields] OR functuional[All Fields] OR functures[All Fields]))
